# Cell quiescence in planarian stem cells, interplay between p53 and nutritional stimuli

**DOI:** 10.1098/rsob.220216

**Published:** 2022-12-21

**Authors:** Gaetana Gambino, Paola Iacopetti, Patrizia Guidi, Chiara Ippolito, Stefania Linsalata, Alessandra Salvetti, Leonardo Rossi

**Affiliations:** ^1^ Department of Clinical and Experimental Medicine, University of Pisa, Via Volta 4, 56126 Pisa, Italy; ^2^ Department of Clinical and Experimental Medicine, University of Pisa, Via Roma 55, 56126 Pisa, Italy; ^3^ Medical Physics Unit, Azienda Ospedaliera Universitaria Pisana, Via Roma 67, 56126 Pisa, Italy

**Keywords:** planarian, stem cells, quiescence, p53, neoblasts

## Abstract

Cell quiescence appeared early in evolution as an adaptive response to adverse conditions (i.e. nutrient depletion). In metazoans, quiescence has been involved in additional processes like tissue homeostasis, which is made possible by the presence of adult stem cells (ASCs). Cell cycle control machinery is a common hub for quiescence entrance, and evidence indicates a role for p53 in establishing the quiescent state of undamaged cells. Mechanisms responsible for waking up quiescent cells remain elusive, and nutritional stimulus, as a legacy of its original role, still appears to be a player in quiescence exit. Planarians, rich in ASCs, represent a suitable system in which we characterized a quiescent population of ASCs, the dorsal midline cord (DMC) cells, exhibiting unique transcriptional features and maintained quiescent by p53 and awakened upon feeding. The function of DMC cells is puzzling and we speculate that DMC cells, despite retaining ancient properties, might represent a functional drift in which quiescence has been recruited to provide evolutionary advantages.

## Graphical abstract

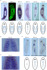

## 1. Introduction

Quiescence is a condition in which cells reversibly arrest their proliferative activity, yet retain the ability to reenter the cell cycle in the presence of appropriate stimuli. Quiescence probably evolved in unicellular organisms as an adaptive response to adverse environmental conditions, especially nutrient depletion, in order to avoid uncoupling of cell division from cell growth, a condition which inevitably compromises cell viability. Thus quiescence appeared early in evolution, spread widely in phylogeny and acquired specialized purposes in diverse biological contexts [1]. Indeed, if, in unicellular organisms, quiescence serves the purpose of aiding the survival of cells until environmental conditions are again suitable for population growth, in rudimentary multicellular species cell quiescence still meets the need of survival, but it also serves the development of novel mechanisms of dissemination. In higher metazoans, proliferating cells reside in a finely controlled microenvironment with constant concentration of cell nutrients, thus releasing cell quiescence from its original role in cell survival and allowing its enrolment in additional purposes, which became fundamental not for single cell survival but for proper tissue homeostasis at organismal level [2]. Tissue homeostasis and regeneration following injury are made possible by the existence of the so-called ‘adult stem cells’ (ASCs) that reside into specialized tissues and are able to proliferate or differentiate according to tissue demand. The balance between quiescence and proliferation significantly affects the growth, maintenance and repair of tissues. In most multicellular organisms, ASCs are in a quiescent state and are specifically activated upon tissue damage to replenish lost tissues or to provide proper turnover of aged cells. In some cases, differentiated cells also lie into a quiescence state, such as for fibroblasts, hepatocytes, lymphocytes and oocytes [3]. Accordingly, cell quiescence plays crucial physiological roles in fundamental aspects of multicellular organism life, including tissue homeostasis and regeneration, immune response and reproduction. To accomplish these functions, fine molecular mechanisms are necessary to regulate the entry and exit from quiescence. Cell cycle arrest is a common hub for different types of quiescent cells; in most cases this takes place prior to S phase, in a resting phase ‘outside of the cell cycle’ called G0, that has a distinct molecular signature from G1 phase observed in cycling cells. Nevertheless, some cases of quiescent cells arrested in the G2 phase of the cell cycle have been described [4–10], a feature that has been associated with efficient tissue regeneration [2]. Quiescence-inducing signals typically act producing a decrease in cyclin-CDK activity or increasing CDK inhibitors or Rb levels; moreover, specific quiescence transcriptional programmes are activated to prevent differentiation, protect cells from accumulating damage over time, inhibit apoptosis. Despite this core molecular machinery, quiescent cells from different origin (i.e. different cell types or cells driven into quiescence through different stimuli) or with differences in quiescence deepness might show substantial heterogeneity in gene expression [3]. A crucial player in maintaining a quiescence status is the p53 protein. Indeed, p53 activity is higher in quiescent cells compared with proliferating cells, and abrogation of p53 function stimulates quiescent normal human fibroblasts to initiate DNA synthesis [11]. A critical role for p53 in regulating quiescence has been also demonstrated in haematopoietic stem cells [12,13] thus suggesting that p53 activation may counteract stem cells expansion through different mechanisms including restriction of self-renewing divisions [14]. Mechanisms responsible for waking up quiescent stem cells remain largely elusive and strictly depend on the specific biological context and the micro environmental conditions. Among these mechanisms the nutritional stimulus, as a legacy of his its original role, still appears to be a key player in quiescence exit. For example, quiescent drosophila neural stem cells are waked by the release of insulin-like peptides by some glial cells following the nutritional stimulus transduced by the fat body [15].

Planarians, Platyhelminthes endowed with remarkable regenerative capabilities, represent a suitable experimentally accessible model system for *in vivo* ASC studies including cell quiescence. Planarian ASCs (historically called the neoblasts) are a heterogeneous population of cells [16,17] constantly recruited to provide new differentiated cells during tissue turn-over and epimorphic regeneration [18], as well as body size growth following feeding [19,20]. Thus, fine regulated mechanisms enhance or decrease planarian ASCs proliferation rate in response to multiple stimuli. Planarian ASCs also represent an adaptive example of divergent regulation of cell cycle check points in which a reduction in the complexity of cell cycle control machinery is emphasized by the loss of an activating E2F transcription factor, cyclin E, and mad1 and mad2, and by the low expression level of CdK2 [21,22]. Here we describe the existence of a quiescent population of ASCs accumulated in a dorsal anterior cord along the midline in the planarian *Dugesia japonica*. ASCs of the dorsal midline show unique transcriptional features and are kept quiescent by the activity of p53 and awakened by nutritional stimulus.

## 2. Results

### 2.1. *Dugesia japonica* has a population of cell cycle-arrested ASCs located in the dorsal midline cord

**Figure 1.**
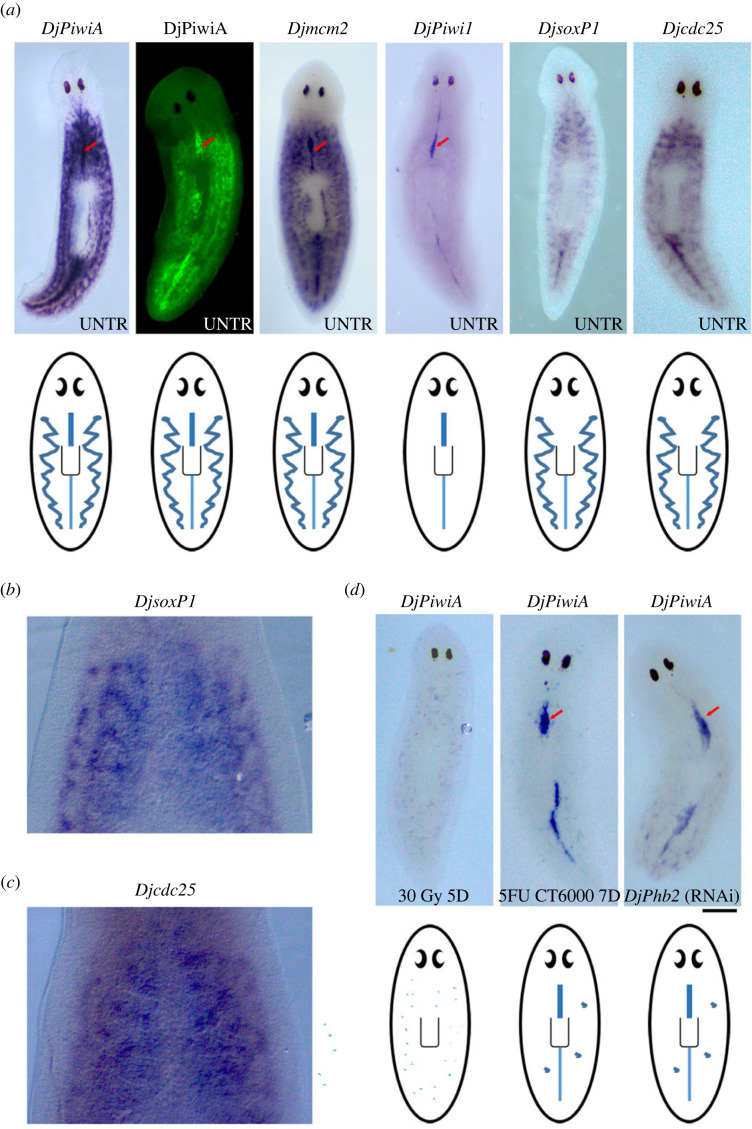
Characterization of DMC cells. (*a*) Image depicting the expression of different stem cell markers in wild-type organisms. In the lower part drawings summarize and schematize the salient features of the different expression patterns. Arrows indicate the dorsal midline cord when stained. (*b*,*c*) Magnification of the planarian body region including the DMC stained by whole mount *in situ* hybridization with *DjsoxP1* (*b*) and *Djcdc25* (*c*) probes, respectively. (*d*) Image depicting the expression of *DjPiwiA* in treated animals. Arrows indicate the dorsal midline cord. Scale bar corresponds to 500 µm in (*a*,*d*) and 100 µm in (*b*,*c*).

**Figure 2.**
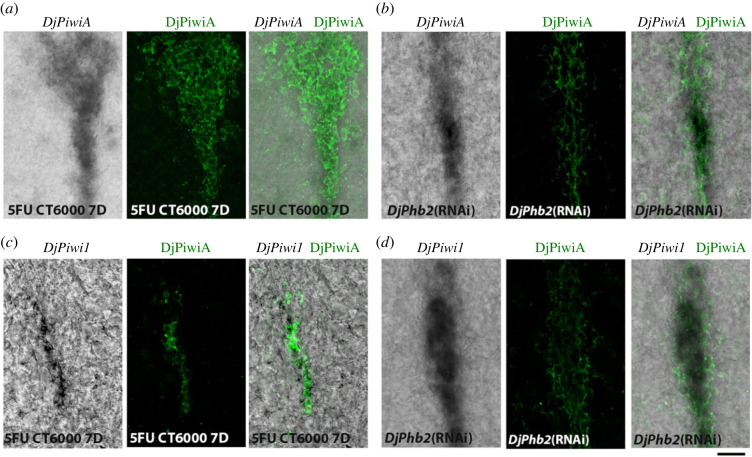
Characterization of DMC cells. (*a*) Co-localization of *DjPiwiA* transcript and protein in DMC cells of a CT6000 animal 7 days after treatment. (*b*) Co-localization of *DjPiwiA* transcript and protein in DMC cells of a *DjPhb2*(RNAi) animal 15 days after first dsRNA feeding. (*c*) Co-localization of *DjPiwi1* transcript and DjPiwiA protein in DMC cells of a CT6000 animal 7 days after treatment. (*d*) Co-localization of *DjPiwi1* transcript and DjPiwiA protein in DMC cells of a *DjPhb2*(RNAi) animal 15 days after first dsRNA feeding. Scale bar corresponds to 25 µm.

The expression of *Smedwi-1*, the transcript coding for PIWI-1 protein of the planarian *Schmidtea mediterranea*, is currently recognized in planarian field, as the standard marker to identify a cell as an ASC and to analyse ASCs distribution in the planarian body [23,24]. *Smedwi-1* expression reveals that ASCs are uniformly distributed through the planarian body, with exception of the pharynx and the anterior part of the head, in a ‘dispersed’ pattern with maximal accumulation close to the gut brunches [25]. The expression of *DjPiwiA* [26], the *D. japonica* orthologue of *Smedwi-1*, reveals that in this species, beside to dispersed neoblasts, dense aggregates of *DjPiwiA*-positive cells are present in a dorsal midline cord (DMC) that runs from behind the eyes to the tail tip, and in some dorso-lateral clusters (figure 1*a*). DMC cells are also identifiable analysing DjPiwiA protein expression (figure 1*a*) and by the expression of other stem cell related genes such as those implicated in DNA replication including *DjMCM2* ([27] and figure 1*a*) and DjPCNA [28]. On the other side, DMC cells show a peculiar molecular signature, indeed, they are the only cells in which the expression of *DjPiwi-1* [29], a fifth PIWI gene that has been only found in Dugesiidae genome and absent in *S. mediterranea* and *Girardia dorotocephala* [30], is enriched above the whole mount *in situ* hybridization detection limit (figure 1*a*). Moreover, DCM cells did not express (at least above the *in situ* hybridization detection limit): (i) *DjsoxP1* (figure 1*a*,*b*), the *D. japonica* homologue of *Smedsox-P1*, one of the markers for neoblast sub-population including the so-called ‘clonogenic neoblasts' [17,31]; (ii) the *D. japonica* cdc25 transcript (*Djcdc25;* figure 1*a*,*c*) and (iii) the tetraspanin group specific gene 1 (*Djtgs-1*) [32], a further putative clonogenic neoblast marker [33]. Similarly to dispersed neoblasts, DMC cells are highly sensitive to sterilizing X-ray doses of 30 Grey (Gy), and completely disappeared 1 day after irradiation [29], a treatment used to identify a cell as a stem cell in the planarian field because it specifically removes stem cells and their progeny following a specific temporal kinetic [25,34]. Five days after irradiation some *DjPiwiA*-positive cells are still detectable in planarian body region unrelated to the DMC (figure 1*d*). Strikingly, DCM cells are resistant to the treatment with high doses of the genotoxicant 5-fluorouracyl (5FU) (5FU CT6000) [35,36] that specifically affect cells during their transit through the S phase of the cell cycle [37], thus suggesting that DMC cells are someway arrested or cycle at a very slow rate (figure 1*d*). Another scenario in which DMC cells show higher resistance with respect to dispersed neoblasts is following the silencing of the prohibitin 2 homologue *DjPhb2* [38]. Indeed, in *DjPhb2*(RNAi) animals 15 days after treatment, *DjPiwiA* expression is only detectable in DMC cells and in some of the dorso-lateral clusters (figure 1*d*). *DjPhb2* is expressed in DMC cells and its silencing produces mitochondrial cristae disorganization and deregulation of mitochondrial transmembrane potential [38]. Thus, the resistance of DMC cells to *DjPhb2* silencing might be explained by a peculiar metabolic condition, in which mitochondria do not play a relevant role in energy metabolism. This feature prompts us to think that DMC cells are ASCs in a quiescent state that, accordingly to what is described for other quiescent ASCs, might be characterized by a specific metabolic signature, that is: they metabolize glucose through glycolysis and the produced pyruvate, instead of being transported into mitochondria, and incorporated into the mitochondrial tricarboxylic acid (TCA) cycle, is transformed to lactate in cell cytosol [39]. In 5FU CT6000 treated worms, as well as in animals silenced for *DjPhb2* expression, DMC cells are positive for both *DjPiwiA* transcripts and DjPiwiA protein (figure 2*a*,*b*). Thereby, although the existence of some cells expressing only one of these factors cannot be completely ruled out, we confidently used anti-DjPiwiA antibodies to mark DMC cells and verify that after 5FU CT6000 or *DjPhb2*(RNAi) the majority of them are also positive for *DjPiwi-1* expression (figure 2*c*,*d*).

**Figure 3.**
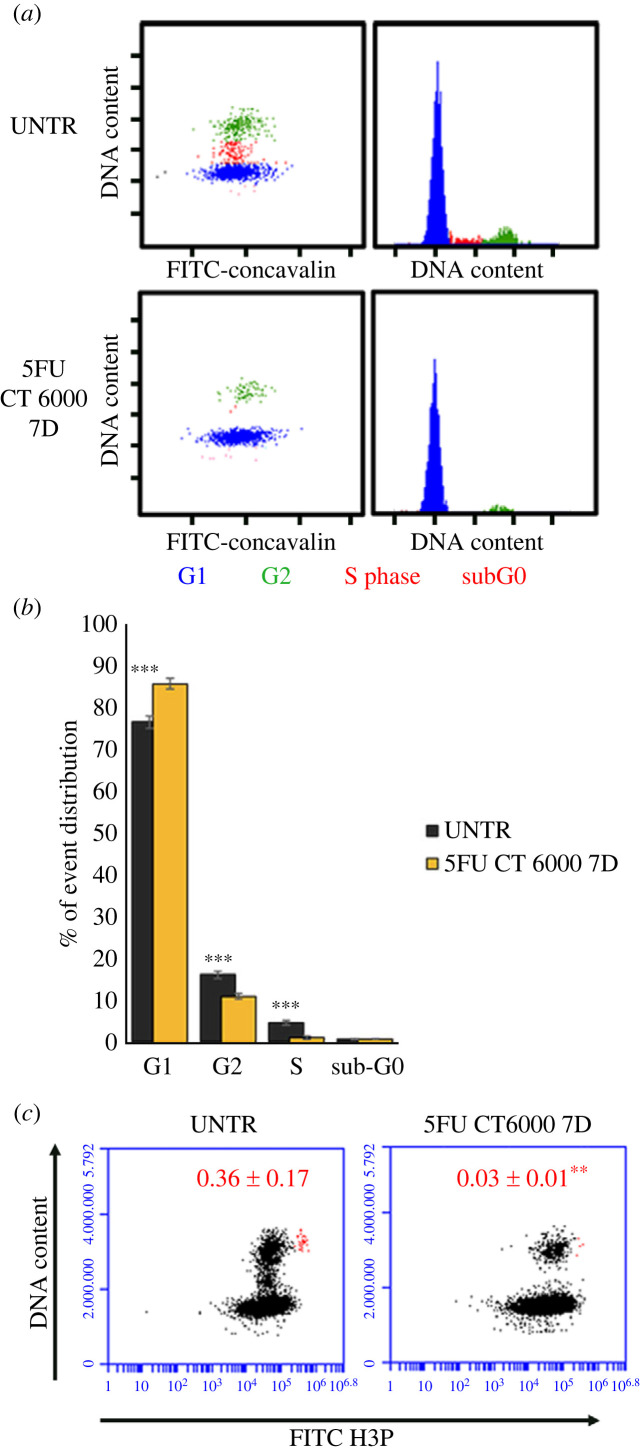
Cell cycle distribution of planarian cells in 5FU CT6000 treated specimens. (*a*) Representative plots depicting the distribution of planarian cells in the cell cycle in untreated controls and 7 days after 5FU CT6000 treatment. (*b*) Graph depicting the distribution of planarian cells in the cell cycle. Each bar is the mean ± s.d. of five independent samples of a representative experiment. (*c*) Representative plots depicting the distribution of planarian cells with regards to DNA content and H3p immunostaining. Cells that were considered mitotic cells are depicted as red dots. Red numbers indicate the mean number of mitotic cells ± s.d. quantified in five independent samples in a representative experiment. **p < 0.01; ***p < 0.005.

**Figure 4.**
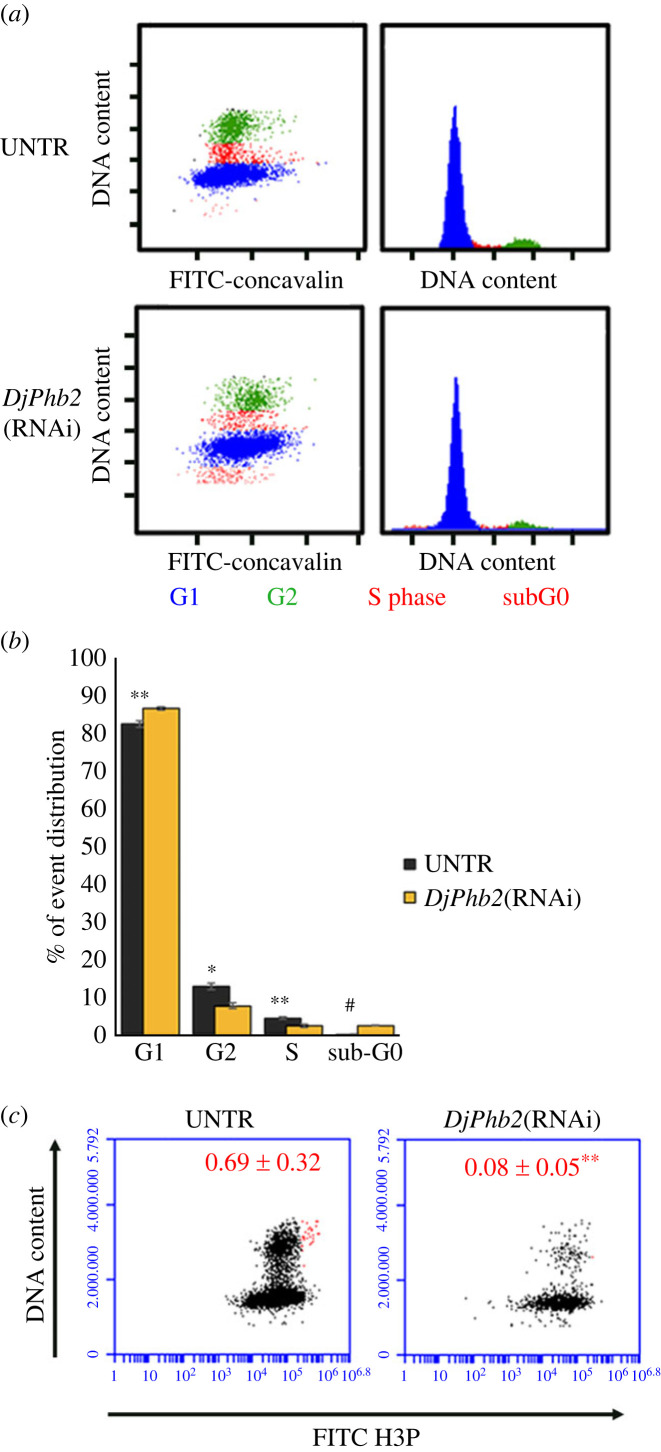
Cell cycle distribution of planarian cells in *DjPhb2*(RNAi) animals. (*a*) Representative plots depicting the distribution of planarian cells in the cell cycle in untreated controls and in *DjPhb2*(RNAi) animals. (*b*) Graph depicting the distribution of planarian cells in the cell cycle. Each bar is the mean ± s.d. of five independent samples of a representative experiment. (*c*) Representative plots depicting the distribution of planarian cells with regards to DNA content and H3p immunostaining. Cells that were considered mitotic cells are depicted as red dots. Red numbers indicate the mean number of mitotic cells ± s.d. quantified in five independent samples in a representative experiment. *p < 0.05; **p < 0.01; ^#^*p* < 0.001.

**Figure 5.**
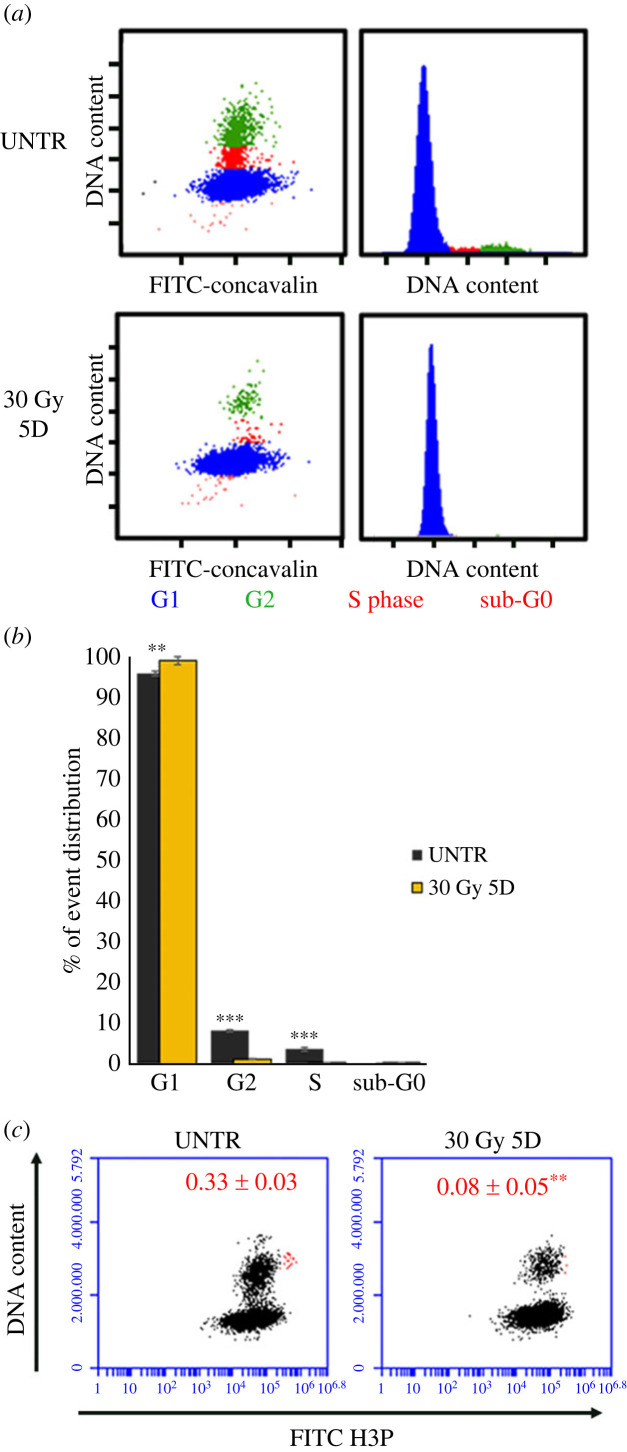
Cell cycle distribution of planarian cells in X-ray treated specimens: (*a*) Representative plots depicting the distribution of planarian cells in the cell cycle in untreated controls and 5 days after 30 Gy X-ray treatment. (*b*) Graph depicting the distribution of planarian cells in the cell cycle. Each bar is the mean ± s.d. of five independent samples of a representative experiment. (*c*) Representative plots depicting the distribution of planarian cells with regards to DNA content and H3p immunostaining. Cells that were considered mitotic cells are depicted as red dots. Red numbers indicate the mean number of mitotic cells ± s.d. quantified in five independent samples in a representative experiment. **p < 0.01; ***p < 0.005.

**Figure 6.**
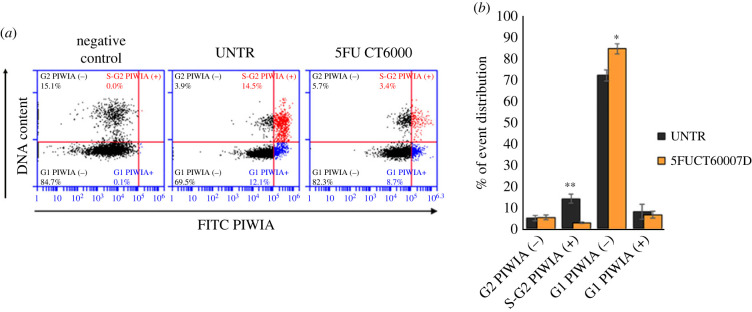
Cytometric quantification of DjPiwiA-positive cells. (*a*) Representative plots depicting the distribution of planarian cells with regards to DNA content and DjPiwiA immunostaining signal. G2 or S phase cells considered positive for DjPiwiA are depicted as red dots; G1 cells considered positive for DjPiwiA are depicted as blue dots. (*b*) Graph depicting the distribution of DjPiwiA cells in the cell cycle. Each bar is the mean ± s.d. of five independent samples of a representative experiment. *p < 0.05; **p < 0.01.

Cell cycle analysis in planarians treated with 5FU CT6000 at the time at which the only detectable *DjPiwiA*-positive cells are almost exclusively the DMC cells, reveals a dramatic drop in S phase cells, a reduction in G2 phase cells and a proportional increase in G1 phase cells (figure 3*a*,*b*). Accordingly, mitoses are almost undetectable (figure 3*c*). However, some rare dividing cells are detectable among DMC cells in challenging conditions after 5FU treatment [35,36], thus allowing to exclude that they are all definitely committed post-mitotic progenies and suggesting that some specific stimuli might trigger, at least for some of them, proliferative activity. Also in the case of *DjPhb2* silencing, a dramatic drop in S phase (figure 4*a*,*b*) and mitosis (figure 4*c*) is detectable, together with a significant decrease in G2 cells, while G1 cells increase proportionally (figure 4*b*). Interestingly, in both *DjPhb2*(RNAi) and 5FU CT6000 as well as after X-ray treatment (figure 5) a proportion of G2 cells remains always detectable (figures 3–5), whose nature, especially 5 days after sterilizing X-ray doses, remains puzzling. As in some planarian species including *D. japonica* mixoploidy (2N, 3N and 4N) has been observed [40], we cannot exclude the possibility that a very small population of 4N cells in the G1 phase of the cell cycle contaminates the G2 peak of 2N cells. However, the comparison in the percentage of remaining G2 cells between 5FU CT6000 (11% ± 0.7), *DjPhb2*(RNAi) (8% ± 0.7) and 30 Gy (1% ± 0.07) specimens clearly indicates that in the first two cases many more G2 cells resist the treatment, suggesting that some of them might be part of the DMC cells. To verify this hypothesis, we investigated cell cycle distribution of DjPiwiA-positive cells by flow cytometry. In untreated controls, this analysis revealed that G2 cells split in two sub-populations, one negative and one positive for DjPiwiA (figure 6*a*,*b*). After 5FU CT6000 treatment, the remaining G2 cells also split in a negative population quantitatively comparable to that of untreated controls, and a positive population (figure 6*a*,*b*) suggesting that at least part of DMC cells are arrested in G2.

### 2.2. Characterization of *Dugesia japonica* p53

**Figure 7.**
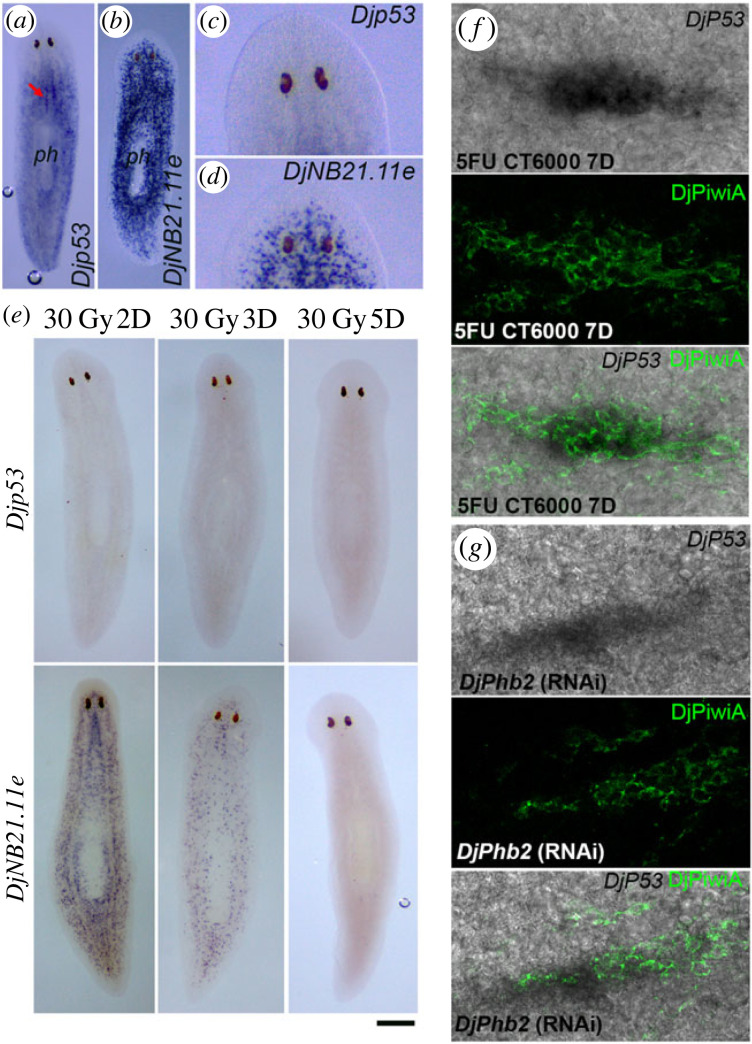
Expression of *Djp53.* (*a*) A representative whole mount *in situ* hybridization image showing the expression of *Djp53* in an untreated worm. Arrow indicates the expression of *Djp53* in DMC cells. (*b*) A representative whole mount *in situ* hybridization image showing the expression of *DjNB21.11e* in an untreated worm. (*c*) Magnification of the head region depicted in (*a*). (*d*) Magnification of the head region depicted in (*b*). (*e*) Representative whole mount *in situ* hybridization images showing the expression of *Djp53* and *DjNB21.11e* at different time points after 30 Gy X-ray treatment. (*f*) Co-localization of *Djp53* transcript and DjPiwiA protein in DMC cells of a CT6000 animal 7 days after treatment. (*g*) Co-localization of *Djp53* transcript and DjPiwiA protein in DMC cells of a *DjPhb2*(RNAi) animal 15 days after first dsRNA feeding. Scale bar corresponds to 500 µm in (*a*,*b*), 100 µm in (*c*), 400 µm in (*e*) and 25 µm in (*f*,*g*).

The importance of p53 protein in the cellular response to DNA damage is well known, however, several lines of evidence suggest that p53 may also play a role in establishing and maintaining the quiescent state of normal undamaged cells to accomplish proper tissue homeostasis [11,13,41,42]. In vertebrates, an essential mechanism for p53 activity regulation is its interaction with the structurally related murine double minute (MDM) proteins 2 and 4 [43]. On the contrary, invertebrates code for only one MDM family protein and, at the moment, a *MDM* gene has been found in only seven invertebrate species, not including Platyhelminthes [44]. Accordingly, in planarians, a MDM-like protein has never been described and our extensive search into genomic and transcriptomic databases did not reveal the existence of a *MDM-like* gene. This allows us to hypothesize that in planarians, p53 might be primarily regulated at the transcriptional level. A planarian homologue of p53 has been described in the species *S. mediterranea*. *Smed-p53* transcript expression pattern mostly overlaps with that of early post-mitotic progeny markers [45]. Differently, despite a basal expression level in post-mitotic progenies cannot be ruled out, the expression pattern of *D. japonica* orthologue of *Smed-p53*, *Djp53*, immediately calls to mind the distribution of ASCs (figure 7*a*). Accordingly, *Djp53* transcripts are accumulated in cells dispersed throughout the mesenchyme, especially close to gut branches, *Djp53* signal is also detectable in DMC cells (figure 7*a*). Moreover, *Djp53* transcripts are not detectable in the pharynx (figure 7*a*) and behind the eyes (figure 7*c*) where the early epidermal post-mitotic progenitors, marked by the expression of *DjNB21.11e* transcripts [46] are widely distributed (figure 7*b*,*d*). Accordingly, *Djp53* expression completely disappears early after 30 Gy X-ray exposure, while, at the same time, the early post-mitotic epidermal progenitors are still detectable (figure 7*e*).

Following 5FU CT6000 treatment or *DjPhb2* silencing, *Djp53* transcripts are still expressed in most of the DMC cells as evidenced by the co-localization with DjPiwiA antibody staining (figure 7*f*,*g*) which, in these experimental conditions, marks the DMC cells. Although significant co-localization between *Djp53* transcript and anti-DjPiwiA antibody is detectable, cells positive for DjPiwiA and negative for *Djp53* and *vice versa* are also observable, suggesting a heterogeneous transcriptional regulation.

*Smed-p53* silencing by RNAi produces a complex phenotype depending on silencing efficiency. Indeed, a silencing by repeated *Smed-p53*-dsRNA feeding induces an initial hyper-proliferation phase in which stem cell number increases at the expense of early progenies, followed by a collapse of the entire stem cell lineage, in which animals lost their stem cell system and die by lysis [45]. In the case of a single RNAi feed, regenerating animals develop some outgrowth at the dorsal surface, but intact animals show no phenotype and escape lysis [45]. The authors reasoned that a lower dose of dsRNA might extend the initial hyperproliferative phase of the phenotype and that the role of Smed-p53 in hyper-proliferation is different from that in stem cell maintenance because at low doses of dsRNA the stem cell population did not deplete [45].

**Figure 8.**
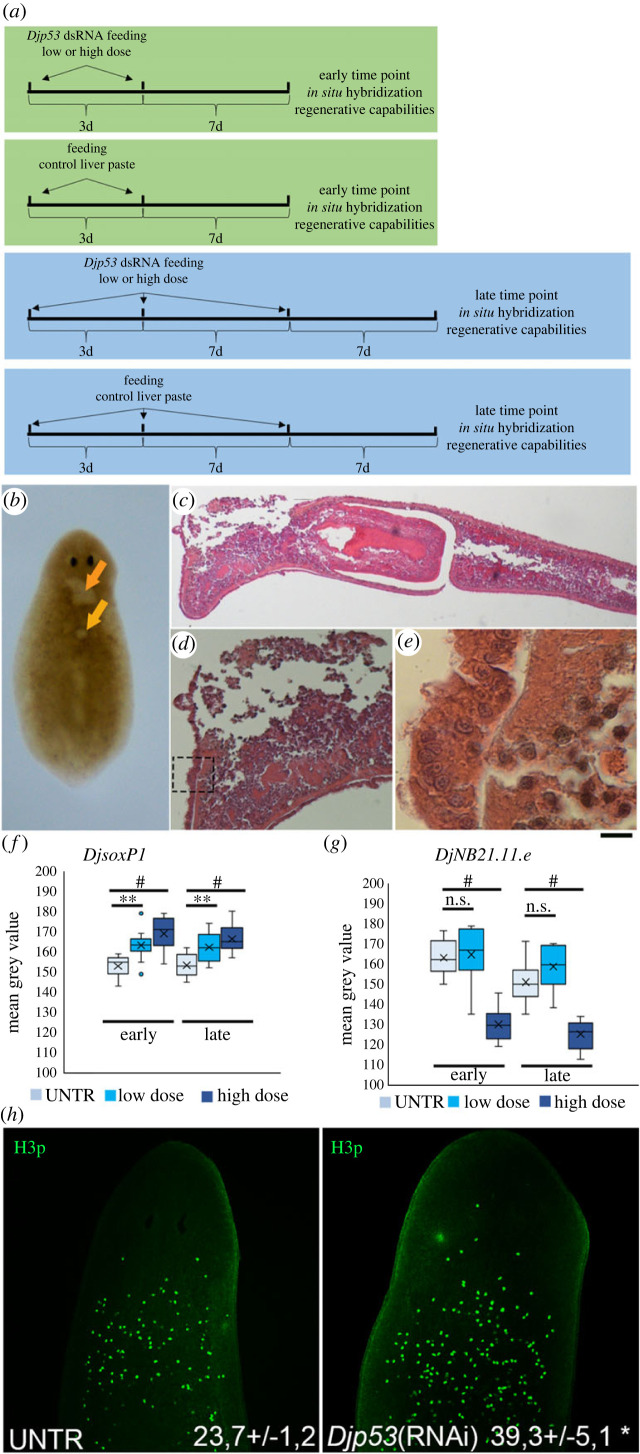
Characterization of *Djp53*(RNAi) phenotype. (*a*) Scheme depicting the experimental design. (*b*) A representative bright field image showing lesions in *Djp53*(RNAi) animals. Arrows indicate lesions. (*c*,*d*) Haematoxylin and eosin stained longitudinal section of a *Djp53*(RNAi) animal showing the histology of a lesion. (*e*) Magnification of the region boxed in (*d*) showing a sub-epidermal blister with cells passing through the basal lamina. Scale bar corresponds to 300 µm in (*b*), 250 µm in (*c*), 60 µm in (*d*) and 25 µm in (*e*). (*f*) Box-plot relating to a representative experiment, depicting the distribution of the expression level (quantified in mean grey value) of the ASC marker *DjsoxP1* in untreated controls and in *Djp53*(RNAi) animals treated with different doses and times. **p < 0.01; ^#^*p* < 0.001. (*g*) Box-plot relating to a representative experiment, depicting the distribution of the expression level (quantified in mean grey value) of the early progeny marker *DjNB22.11.e* in untreated controls and in *Djp53*(RNAi) animals treated with different doses and times. ns: not significant; ^#^*p* < 0.001. (*h*) Representative confocal images showing mitotic cells identified by H3p immunostaining in the anterior region of the planarian body. Numbers indicate the mean number of mitotic cells ± s.d. quantified in five independent samples of a representative experiment. *p < 0.05.

The fact that different dosages or a single feeding versus repeated feeding impact on the phenotype outcome, has made it necessary to carry out preliminar investigation on the effects produced by *Djp53* RNAi in *D. japonica.* To this aim we silenced *Djp53* expression by using high (450 ng µl^−1^ of liver paste corresponding to about 200–300 ng for each animal) or low (150 ng µl^−1^ of liver paste corresponding to about 70–100 ng for each animal) doses of dsRNA, and sacrificed the animals at early and late time points according to the scheme depicted in figure 8*a*. At the late time point (15 days after the first dsRNA administration), dorsal lesions, mainly localized in the anterior part of the body between the pharynx and the auricles, appeared in some RNAi animals in both the low (7/50) and the high (16/50) dose groups (figure 8*b*). At the lesion site, the entire tissue morphology is destroyed and the epidermis is lost or detached (figure 8*c*,*d*). In addition to these large lesions, small epidermal blisters, resembling those described in RNAi phenotypes of the tumour suppressor genes *Smed-Gli pr1* and *Smed-MpB* [47], were detectable (figure 8*e*), thus suggesting that *Djp53* acts as a tumour suppressor gene in *D. japonica* preventing stem cell proliferation outside a proper microenvironment. After lesion occurrence animals died in a few days. On the contrary, early time point animals were alive and appeared healthy. In way to analyse stem cell and early progeny fate in *Djp53*(RNAi) animals, we quantified the expression of *DjsoxP1* and *DjNB21.11e* transcripts. We observed a significant dose dependent increase in *DjsoxP1* signal (figure 8*f*). In addition, by visualizing its expression pattern in a large number of *Djp53*(RNAi) animals, we were able to observe about 33% of specimens with a clearly detectable signal in a territory resembling that of the DMC cells, never observed in untreated control samples (electronic supplementary material, figure S1A). *DjsoxP1* expression was higher that controls also in animals showing dorsal lesions (data not shown). Number of early epidermal progenies was significantly reduced only in high dose treated animals at both early and late time points (figure 8*g*; electronic supplementary material, figure S1B). Finally, we monitored the regenerative capabilities of RNAi animals measuring the blastema area in tail pieces 4 days after experimental amputation was performed at early and late time points after feeding. At the early time point no significant differences were detectable between RNAi animals and corresponding controls, despite a reduction trend was observable for the high dose samples (electronic supplementary material, figure S1C). At the late time point, animals with lesions died a few hours after cut, while organisms without lesions were able to onset a regenerative response; however, animals treated with the high dose produced a blastema significantly smaller than that of control animals (electronic supplementary material, figure S1C).

In order to investigate the role of Djp53 in the regulation of DMC cell activity, we needed to identify the experimental condition which guaranteed a wide temporal window in which the hyperproliferative phase dominated ASC activity, with the least possible impact on animal physiology. For this reason, we discarded the late time point in way to avoid lesions and animal death and we selected for all the following experiments the early point at the high dose. We also analysed the number of mitotic cells by H3p immunostaining, in this experimental condition. As shown in figure 8*h*, the number of mitosis was significantly higher in *Djp53*(RNAi) animals with respect to controls.

### 2.3. p53 restricts proliferative activity of DMC cells

**Figure 9.**
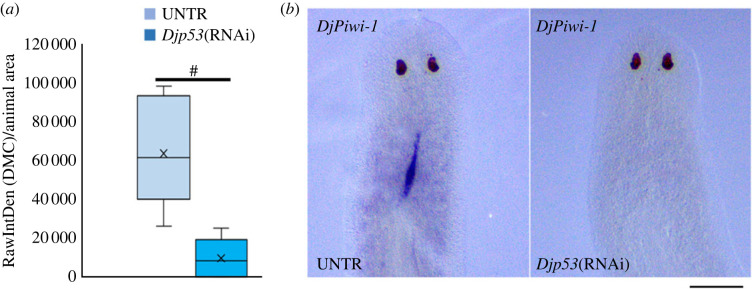
Effect of *Djp53* silencing on *DjPiwi1* expression. (*a*) Box-plot relating to a representative experiment, depicting the distribution of the expression level (quantified in terms of RawIntDen of the DMC/animal area) of *Djpiwi1.* In *Djp53* (RNAi) animals and corresponding controls ^#^*p* < 0.001. (*b*) Representative images of *Djpiwi1* expression in *Djp53*(RNAi) animals and corresponding controls. Scale bar corresponds to 500 µm.

**Figure 10.**
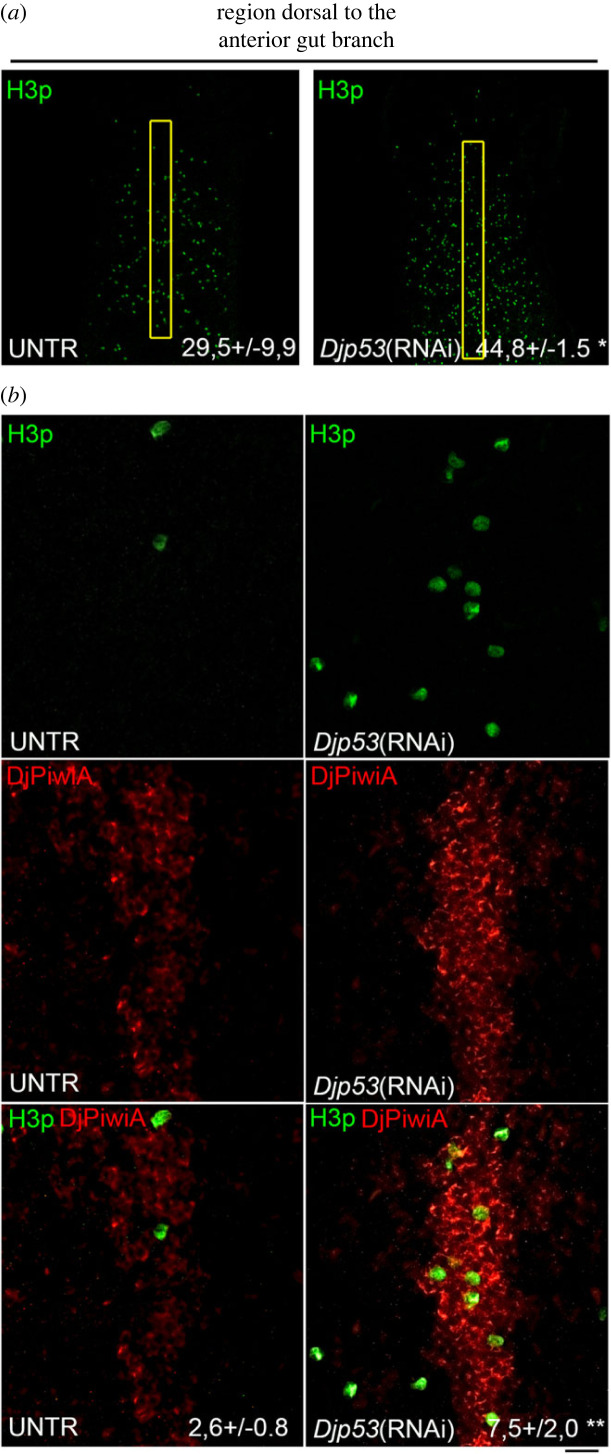
Effect of *Djp53* silencing on mitotic activity in the dorsal midline cord. (*a*) Representative confocal images showing mitotic cells identified by H3p immunostaining in the anterior dorsal region of the planarian body. The yellow boxes correspond to the region delimited for quantification of H3p-positive cells. The mean number of mitotic cells ± s.d. quantified in five independent samples of a representative experiment is indicated. (*b*) Representative images of DjpiwiA and H3p co-immunolabelling in an untreated control and in a planarian silenced for *Djp53* expression. The mean number of mitotic cells ± s.d. quantified in five independent samples of a representative experiment is indicated.*p < 0.05; **p < 0.01. Scale bar corresponds to 200 µm in (*a*) and 20 µm in (*b*).

**Figure 11.**
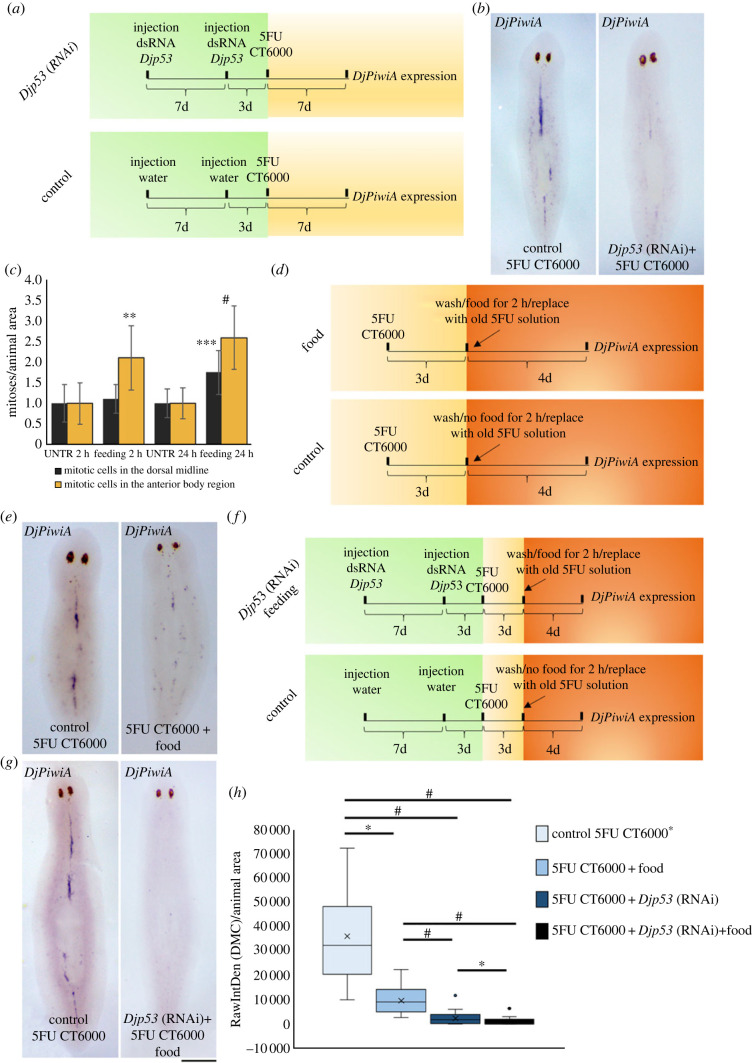
Effect of *Djp53* silencing, feeding and their combination on DMC cells. (*a*) Scheme depicting the experimental design to investigate the effect of *Djp53* silencing on DMC cells. d = days. (*b*) Representative images of whole mount *in situ* hybridization showing the effect of *Djp53* silencing on *DjPiwiA*-positive cells following 5FU CT6000 treatment. (*c*) Graph depicting the number of mitotic cells counted 2 and 24 h after feeding in the anterior body region or in the presumptive territory including DMC cells. Each bar is the mean value ± s.d. of five independent animals. **p < 0.01; ***p < 0.005; ^#^*p* < 0.001. (*d*) Scheme depicting the experimental design to investigate the effect of feeding on DMC cells. d = days. (*e*) Representative images of whole mount *in situ* hybridization showing the effect of feeding on *DjPiwiA*-positive cells following 5FU CT6000 treatment. (*f*) Scheme depicting the experimental design to investigate the effect of *Djp53* silencing and feeding on DMC cells. d = days. (*g*) Representative images of whole mount *in situ* hybridization showing the combined effect of *Djp53* silencing and feeding on *DjPiwiA*-positive cells following 5FU CT6000 treatment. (*h*) Box-plot relating to a summary experiment, depicting the distribution of the expression level of *DjPiwiA* in DMC cells (in terms of RawIntDensity normalized versus animal size) following 5FU CT6000 treatment alone or in combination with *Djp53* silencing, feeding or a combination of both. Asterisk in the label indicate that the control is the most complete including water injection as depicted in figure 6*f*. *p < 0.05; ^#^*p* < 0.001. Scale bar corresponds to 500 µm in (*g*) and 600 µm in (*b*,*e*).

To specifically analyse the effect of *Djp53* silencing on DMC cells we firstly decided to analyse *DjPiwi-1* expression in RNAi animals. Surprisingly, we found that *DjPiwi-1* signal in the DMC dramatically drops (figure 9*a*), to the point of becoming undetectable in DMC cells of some *Djp53*(RNAi) animals (figure 9*b*). This, together with the appearance of *DjsoxP1* signal in a territory resembling DMC cells following *Djp53* silencing (electronic supplementary material, figure S1A), suggests an involvement of p53 in modulating the transcriptional profile of DMC cells. Moreover, its expression in DMC cells after 5FU CT6000 treatment (figure 7*f*) [35], suggests a role for this transcription factor in maintaining these cells arrested in the cell cycle. To verify this possibility, we first grossly proved that following *Djp53* silencing, in a context of a generalized increase in proliferation, a significant increase in mitosis is also observable in the body region corresponding to the median dorsal cord (figure 10*a*) and we confirmed this data by counting mitosis number in the DMC identified by anti-DjPiwiA antibody labelling (figure 10*b*). These findings pushed us to design specific experiments to emphasize the role of p53 in the control of DMC cells without the general nuisance of all the other stem cells of the planarian body. To this aim, we silenced *Djp53* by injecting dsRNA molecules at a dose (300 ng animal^−1^) corresponding to the high dose delivered by feeding and then treated the animals with 5FU CT6000 (following the experimental scheme depicted in figure 11*a*). We reasoned that whether DMC cells normally resist to 5FU treatment due to their low proliferative activity, in case p53 is responsible for their arrest in the cell cycle, its silencing will release these cell from quiescence thus making them sensitive to the genotoxic drug. If this is the case, we should observe a reduction in *DjPiwiA* signal marking the DMC. As shown in figure 11*b*,*h*, *DjPiwiA* signal is strongly reduced in *Djp53* (RNAi) + 5FU CT6000 animals with respect to 5FU CT6000 animals.

### 2.4. DMC cells are released from quiescence following a nutritional stimulus

If we allow that p53 might play an essential role in preventing DMC cell proliferation, we also wonder which signals might be responsible for DCM cells activation and reentrance into the cell cycle. It is well known that planarian ASCs are immediately induced to divide following feeding [19]. This observation makes us interested in verifying whether nutritional stimulus might, in parallel to a general propulsion for ASC proliferation, be a potent effector also for the activation of DMC cells. To this aim, we firstly analysed the distribution of mitotic events in the dorsal anterior region of intact planarians at 2 and 24 h after feeding. We found the expected increase in mitotic cells in the entire scanned region at both analysed time points (figure 11*c*). Restricting our analysis only to the dorsal midline, a significant increase can be observed exclusively 24 h after feeding (figure 11*c*). We confirmed this data counting mitosis number in the DMC identified by anti-DjPiwiA antibody labelling (electronic supplementary material, figure S2). Pushed by this preliminary observation we designed a specific experiment to focus our attention on the DMC cells. Hypothesizing that a nutritional stimulus activates DMC cells to proliferate, feeding under a 5FU CT6000 regimen should wake DMC cells from quiescence, induce them to proliferate thus making them more sensitive to the genotoxic drug. To this aim we treated the animals with 5FU CT6000 for 3 days, then we changed the medium with fresh planarian water, kept aside the 5FU solution, and fed the animals for a couple of hours. Following feeding, animals were transferred back to the old 5FU solution for additional 4 days (figure 11*d*). As shown in figure 6*e*,*h*, the administration of food under the CT6000 regimen produces a strong significant reduction of *DjPiwiA*-positive DMC cells. To further prove our finding we reasoned that the concomitance of *Djp53* silencing and the administration of a nutritional stimulus might produce a synergistic effect in stimulating DMC cells proliferation. Thus, we treated *Djp53* silenced animals with 5FU CT6000 and followed the previously described feeding procedure (figure 11*f*). Accordingly to what was expected, a dramatic reduction in *DjPiwiA* signal at the level of DMC was observed with almost all the animals with no trace of hybridization signal in the DMC especially anterior to the pharynx (figure 11*g*,*h*).

## 3. Discussion

Regulation of cell proliferation rate is fundamental for proper tissue homeostasis and regulation of body size. ASC quiescence allows the maintenance of a pristine population of stem cells into adult tissues ready for a prompt activation in case of need; in the choice whether to exit or not the quiescent state ASCs integrate a variety of local and systemic signals including hormones, growth factors and metabolic mediators. Quiescence exit is also heterogeneous: following the same signal, some cells reenter the cell cycle while others remain quiescent thus avoiding the complete exhaustion of a pool of quiescent cells with a single stimulus [48]. Despite the fundamental role of quiescence in controlling animal growth, as well as its relevance as a therapeutic challenge, being for example the quiescent state of the ‘cancer stem cells’ a protective condition against antiproliferative agents [49], mechanisms controlling the switch from proliferation to quiescence and *vice versa*, as well as those underlying the heterogeneity in quiescence exit are poorly understood [3]. Planarians, with their large population of experimentally accessible ASCs are an ideal model system to identify mechanisms responsible for the control of proliferation rate. In particular, organisms belonging to the *D. japonica* species offer the possibility to study the regulation of a spatially identifiable population of quiescent ASCs (i.e. the DMC cells). These cells can be reasonably considered as a part of the complex neoblast system. Indeed, they express DNA replication factors, standard markers used to identify a planarian cell as a stem cell, are sensitive to sterilizing X-ray doses and can be also induced to proliferate in challenging conditions. Nevertheless, DMC cells show a specific molecular signature, survive in condition in which mitochondrial cristae morphogenesis is compromised and are resistant to high doses of the genotoxicant 5FU, strongly suggesting that most of them progress through the cell cycle very slowly or do not enter the S phase.

Planarian ASCs modulate their proliferative activity in dependence of several stimuli: (i) during the regeneration process in which two impressive proliferative waves produce a regenerative blastema for epimorfic reconstruction of missing tissues [18,50]; (ii) during the repopulation process following sub-lethal doses of X-rays [51] or a single dose treatment with 5FU [36]; and, finally, (iii) in response to feeding [19]. Indeed, planarians change their body size depending on the nutritional status: feeding induces ASC proliferation that results in an increase in cell number and in animal growth, while following starvation, the number of cells decreases and body size shrinks leading to de-growth [52,53], suggesting that energy metabolism is a central cue for proliferation control, thus linking the control of cell quiescence to its original evolutionary origin in unicellular organisms. DMC cells are not significantly activated during the regeneration process [18,29], neither their poor proliferative activity seems to play an essential role in the repopulation process following ASC partial depletion [36]. On the contrary, here we demonstrate that these cells respond to nutritional stimuli, not as a direct consequence of intestinal filling but belatedly, suggesting the intermediation of some still unknown molecular signal. Indeed, as a consequence of feeding most of DMCs cells became sensitive to 5FU treatment, while some still resist, suggesting a certain level of heterogeneity in the dormancy level and in the response to food. The identification of specific molecular pathways linking nutrient supply with quiescence exit of DMC will be the next challenge to face. If on one side, the food supply is able to awake DMC cells, their quiescent status seems to be directly related to p53 expression level. The idea to focus our attention on p53 came from the evidence that in *D. japonica* this gene is mainly and stably expressed in neoblasts, including the DMC cells which also express this gene later after 5FU treatment or *DjPhb2*(RNAi) [35] (and data provided in this paper). The release of p53 constrain produces *D. japonica* ASC hyper-proliferation and gradual reduction in early post-mitotic progenies, leading in some cases, to the development of dorsal lesions and epidermal blisters. Only some aspects of the *Smed-p53* (RNAi) phenotype were recapitulated in *D. japonica* in our hands. Indeed, despite repeated feeding and high doses, the stem cell system never collapsed until animal death caused by lesions, and in most cases animals maintain regenerative capabilities. Differences between *Smedp53* and *Djp53* in both expression territories and RNAi phenotype suggest that in *D. japonica* this gene is mainly involved in modulating stem cell progression through the cell cycle with a marginal role as a cell fate switcher in the transition from immediate stem cell daughters to early progenies as suggested for *Smed-p53* [45], thus leading us to hypothesize that in dependence on its abundance this gene might balance ASCs fate, governing their proliferation rate/differentiation propensity. p53 silencing increases DMC cell sensitivity to 5FU treatment demonstrating that p53 expression is sufficient to maintain these cells in a non-proliferative status; also in this case, not all DMC cells respond to p53 silencing in the same way further evidencing a certain level of heterogeneity. Consistent with these results is the change in the transcriptional profile of DMC cells produced by p53 silencing. Indeed, DMC cells of *Djp53*(RNAi) animals became, in some cases, positive for *Djsox-P1* and dramatically downregulate *DjPiwi-1* expression. In this view, *DjPiwi-1* expression level appears to correlate with a specific ASC fate as further suggested by its transient upregulation outside the canonical DMC domain during wide repopulation processes such as following sub-lethal X-ray or 5FU treatments [36,51]. *DjPiwi-1* silencing did not produce significant phenotypes in physiological conditions [29], however, further future insights into challenging contexts might reveal a still unknown function for this gene. The concomitant p53 knockdown and food supply synergize in promoting DMC cell entrance in the cell cycle. The crosstalk between p53 and food supply that we demonstrated in DMC cells, could be theoretically generalized to all planarian ASCs, and we can imagine that in a physiological context the activity of p53 and nutritional stimuli antagonize themselves in controlling ASC fate, suggesting a simple two-factor model in which signals produced following nutritional stimuli overcome p53 constrain thus pushing neoblasts toward proliferation with consequent increase in cell number and animal size. As nutritional stimuli gradually decrease, p53 constrain is re-established thus inducing the differentiation of part of the newly produced stem cells; in this way the ratio between stem cells and differentiated cells slightly decreases with animal growth as previously demonstrated [19]. In the case of prolonged starvation nutritional stimulus is totally absent, however, it is well known that long starvation periods do not cause a decrease or an increase in the mitotic rate, which remains constant to a ‘basic mitotic index’ [20], suggesting a mechanism that despite the absence of nutrients allows for proper cell turn-over and tissue homeostasis. A possibility might be a differential regulation of p53 activity (i.e. at transcriptional level), a condition that makes ASCs more apt to proliferate despite the shortage in nutrients. Further experiment in this direction might highlight a link between p53 and nutritional stimuli.

Despite the advances in the characterization of DMC cells, their function in the physiology and homeostasis of *D. japonica* remains obscure. These cells are indeed dispensable for all the planarian biological performances, and a genetic strategy aimed to specifically eliminate them, leaving unaltered all the other neoblasts, has not yet been found, thus preventing the identification of DMC cell specific role. For example, the long-term silencing of their specific marker *DjPiwi1* does not produce a phenotype not alter DMC distribution/abundance (data not shown). The specific anatomical position of DMC cells does not correspond to specific differentiated structures, thus reinforcing the idea that these cells are not progenitors for a specific cell lineage. Moreover, a similar accumulation of ASCs with these properties is not detectable in *S. mediterranea*, despite the existence of a slow-cycling population of TOR-neoblasts (the RNA-low neoblasts), has been recently identified [54]. Evolution does not take missteps and since the state of quiescence of the DMC cells is firmly maintained, they should constitute an advantage for *D. japonica*. A very audacious speculation about a putative function for these cells came from the reading of the unpublished pre-print of Davidian *et al.* [55] in which they demonstrate the ability of electric stimulation to reverse damaging effects of high dose X-rays inducing ectopic expression of stem cell markers in post-mitotic cells. However, this phenomenon is only observable when in the irradiated host is grafted a small piece of tissue from a donor containing neoblasts. The authors unequivocally demonstrate that neoblasts of the grafted tissues are not those reappearing in the irradiated host tissue, but no suggestions are made to explain the necessity of neoblasts in transplanted tissue for electric stimulation-mediated expression of neoblast markers. According to these findings it might be thought that the presence of some neoblasts in the planarian body is someway indispensable for promoting the plasticity of post-mitotic progenitors to revert their differentiation process and provide novel stem cells when the animals face very challenging conditions in which most stem cells are lost. In this scenario DMC cells, which in accordance with their quiescent status are resistant to several environmental insults, might represent a reservoir of stem cells distributed along the entire animal length that mediate (hypothetically by releasing molecular signals) the interplay among ASCs, progenitors and post-mitotic progenies. In this view quiescence of DMC cells, despite retaining ancient properties (i.e. dependence on food supply), might represent a functional drift in which the same basic cellular process has been reprocessed in a multicellular context to provide an evolutionary advantage.

## 4. Material and methods

### 4.1. Planarian rearing, 5FU treatment, X-ray treatment and morphometric analysis of blastema size

Planarians belonging to the species *D. japonica* asexual strain GI [56] were reared in artificial water as described by Cassella *et al.* [46]. Animals were fed on chicken liver and starved for two weeks before the experiments, unless otherwise specified. For blastemal size measurement, 30 planarians for each experimental class were cut between the auricles and the pharynx, left to regenerate for 4 days and then killed in 2N hydrochloric acid for 5 min at 4°C, fixed in 4% formalin in PBS plus 0.1% triton-X 100 (PBST-01) for 20 min, briefly washed in PBST-01 and depicted under a Zeiss stereomicroscope (Stemi 305, Carl Zeiss Microscopy GmbH) equipped with a digital camera (AxiocamErc 5s, Carl Zeiss Microscopy GmbH) using the Zeiss acquisition software AxioVision SE64 Rel. 4.9.1. Blastema and animal size of tail fragments were then measured using the ImageJ software [57]. We considered as blastema the unpigmented region below the wound epithelium; blastemal margin was manually highlighted by the operator in blind. As described in Salvetti *et al.* [58], in order to normalize differences in blastema area that might depend on the use of animals of different sizes, we chose the ratio between blastema area and total body area as the most appropriate parameter to evaluate effects on tissue regeneration. 5FU was purchased from Sigma (F6627, Sigma-Aldrich) and diluted in DMSO to obtain a 400 mM stock solution. Treatments were performed in **C**ontinuous **T**reatment regimen with 6000 µM of 5FU (CT6000), as described in [35]. For irradiation experiments, planarians were exposed to a single fraction dose of 30 Gy (uncertainty ± 2%) using a 15 MV X-rays beam at dose rate of 6 Gy min^−1^ on a Varian Medical System Clinac DHX linear accelerator for radiotherapy.

### 4.2. RNA interference

Double-stranded RNAs (dsRNA) of *Djp53* was produced by *in vitro* transcription of the corresponding template, obtained by RT-PCR on wild-type planarian cDNA, with the following T7 promoter adapted forward and reverse primers, respectively: tataatacgactcactatagggGGCACAGCAGTATATCACAACA; tataatacgactcactatagggAATACATTGAGCTTTTTCTGGATCAC. RNA interference (RNAi) was performed both by using the feeding procedure as described in Rossi *et al.* [59] using 150 ng µl^−1^ or 450 ng µl^−1^ for low and high dose, respectively, and by the injection procedure by using a Nanoject II Auto-Nanoliter Injector Automated (Drummond Scientific Company). In this last case about 300 ng of dsRNA were injected into single animals each week.

### 4.3. Whole mount *in situ* hybridization and whole mount fluorescent immunostaining

DNA templates for *DjPiwiA* and *DjNB.21.11.e* were prepared by RT-PCR from wild-type planarian cDNA, using T7 promoter adapted primers, as previously described by Cassella *et al*. [46]. *DjPhb2* DNA template was obtained as described by Rossi *et al.* [38]. *DjsoxP1* and *Djp53* DNA templates were obtained as described in [35]. *DjMCM2*, *DjPiwi1* and *Djcdc25* DNA templates were obtained by RT-PCR from wild-type planarian cDNA by using the following T7 promoter adapted primers:

*DjMCM2* forward: GAGCAATAATTTGGAACGAAGTC

*DjMCM2* reverse: CGGATATAATACGACTCACTATAGGGCGGATATAATACGACTCACTATAGGGTCCC

*DjPiwi1* forward: CAACCTGCAAATTATTATGGGTCAAG

*DjPiwi1* reverse: CGGATATAATACGACTCACTATAGGGCGGATATAATACGACTCACTATAGGGACTT

*Djcdc25* forward: TCCAGACCAGTAGTAGAGACTACACGTTATTTCGCAACTCCTGGT

*Djcdc25* reverse: CGGATATAATACGACTCACTATAGGGTGTCCAGTGTGTGATTGCCT

The sequence for *D. japonica* homologue of *S. mediterranea cdc25* was identified by BLAST searching in *D. japonica* transcriptome (dd_Djap_v4) available in the PLANMINE database [60]. Purified amplification products were *in vitro* transcribed in the presence of digoxigenin (DIG)-labelling mix (11277073910, Roche) to obtain DIG-labelled RNA probes.

*In situ* hybridization procedure was performed according to the protocol described by Cassella *et al.* [46]. For semi-quantitative experiments, single specimens were depicted in the same illumination, exposure and acquisition conditions under a Zeiss stereomicroscope (Stemi 305, Carl Zeiss Microscopy GmbH) by using a digital camera (AxiocamErc 5s, Carl Zeiss Microscopy GmbH) and the Zeiss acquisition software AxioVision SE64 Rel. 4.9.1. Phospho-histone H3 (Ser10) (H3p) and DjPiwiA [61] immunostaining was performed following the protocol described by Cassella *et al.* [46] and Rossi *et al.* [38], respectively. Double immunostaining with both antibodies was performed following the protocol described by Cassella *et al.* [46]. *DjPiwiA*, *DjPiwi-1* and *Djp53* RNA and DjPiwiA protein co-localization was performed by staining the animals with anti-DjPiwiA antibody following hybridization with RNA probes. At the end of immunostaining procedure, specimens were mounted in 80% glycerol and scanned under a TCS SP8 confocal microscope (Leica Microsystems CMS) by zeta stack optical sectioning every 2 µm and tile scan function when needed.

### 4.4. Cytofluorimetry

For cytofluorimetry analysis we followed the ACME fixation/dissociation procedure described by García-Castro *et al.* [62]. Analysis of cell cycle was performed by staining the cytoplasm of dissociated/fixed cells with Concanavalin-A conjugated with Alexa Fluor 488 (C11252, Invitrogen by Thermo Fisher Scientific), following the protocol described by García-Castro *et al.* [62]. Cell DNA was stained with 1 mg ml^−1^ propidium iodide (PI) (P1304MP, Invitrogen by Thermo Fisher Scientific) following RNA digestion. In details, dissociated/fixed cells were suspended in PBS plus 1% bovine serum albumin (PBS-BSA), and after RNA digestion with 50 µg ml^−1^ DNAse-free RNAse A (EN0531, Invitrogen by Thermo Fisher Scientific) for 30 min at 37°C, PI was added to the cell suspension and incubated for 45 min in the dark before running into the cytometer. Stained cells were visualized using an ACCURI C6 PLUS (BD Biosciences) cytofluorimeter. Doublets discrimination was manually performed by plotting events on a linear scale according to FSC signal area versus FSC signal height. Gated events were then visualized in linear scale according to FL3 670 LP channel signal area versus FL3 670 LP channel signal height, in this way a second gating was produced to eliminate debris with very low PI staining. Gated events were finally plotted in FL3 670 LP channel (linear) versus FL1 533/30 channel (logarithmic), or in channel FL3 670 LP (linear) to produce a graph depicting the distribution of planarian cells in the cell cycle. A minimum of 5000 events were acquired for each sample. For the detection of H3p-positive cells, fixed/dissociated cells were suspended in PBS-BSA plus 0.1% triton X-100 for 15 min. Cells were collected by centrifugation at 1200 r.p.m. for 5 min at 4°C and suspended in PBS-BSA containing anti-pS10H3 FLUO antibody (1 : 50 dilution) (06–570-AF488 Merck, EMD Millipore Corp.) for 2 h at room temperature. After two washes with PBS-BSA, cells were stained with PI and a minimum of 25 000 events were recorded. For the detection of DjPiwiA-positive cells, fixed/dissociated cells permeabilized with triton X-100 as previously described, were incubated in 1 : 100 dilution of mouse anti Djpiwi A (a kind gift of professor Agata Kyokazu, National Institute for Basic Biology, Japan) for 2 h at room temperature. After a wash in PBS-BSA, cells were incubated with a 1 : 200 dilution of secondary anti-mouse antibodies conjugated with Alexa-fluor 488 (Invitrogen by Thermo Fisher Scientific) for 30 min at room temperature in the dark. After a wash in PBS-BSA, cells were stained with PI and a minimum of 10 000 events were recorded.

### 4.5. Image managing, data analysis and statistics

#### 4.5.1. Qualitative image processing

Representative images not subjected to quantification analysis have been minimally processed modifying brightness/contrast balance, or changing the colour balance to standardize aesthetic presentation without misrepresenting any information of the original picture.

#### 4.5.2. Quantification of labelling signal intensity in whole mount *in situ* hybridization experiments

Hybridization signal intensity was quantified using ImageJ software [57]. Two different approaches were followed in way to perform a semi-quantitative measurement of signal intensity: (i) for the quantification of *DjsoxP1* and *Djcdc25* signal, single pictures were converted in greyscale 8-bit images and inverted. The depicted animal was then manually selected and the mean grey value (sum of the intensity of each single pixel included in selection/total number of pixels) was quantified and used for data analysis and statistical evaluation considering each single animal as an independent sample. Two independent experiments were performed for each experimental dataset to verify the consistency of the results, only one of the two independent experiments is shown. The non-parametric, two-tailed Mann–Whitney *U*-test was applied across each experiment (*n* = 30) to analyse the statistical significance of differences between each treatment group and the untreated control group. Differences were considered statistically significant with a *p*-value below 0.05. (ii) For the evaluation of the *DjPiwiA or DjPiwi1*-stained DMC cells, in way to consider both intensity and amplitude of signal, single pictures were converted in greyscale 8-bit images, then a manual threshold was introduced to select stained DMC cells in the anterior part of the animal. The threshold was adjusted in way to ensure the selection of the DMC in all control samples and then maintained constant throughout all the datasets. In the selected area, the RawIntDensity (sum of the intensity of each single pixel included in selection) was recorded and then normalized versus the entire animal area, in way to eliminate differences in DMC dependent on animal size. Normalized data were used for statistical evaluation considering each single animal as an independent sample. Two independent experiments were performed for each experimental dataset to verify the consistency of the results, only one of the two independent experiments is shown. The non-parametric, two-tailed Mann–Whitney *U*-test was applied across each experiment (*n* = 30) to analyse the statistical significance of differences between each treatment group and the untreated control group. Differences were considered statistically significant with a *p*-value below 0.05.

#### 4.5.3. Distribution of planarian cells in the cell cycle phase

In way to identify the distribution of cells in the different phases of the cell cycle linear plot of PI staining intensity were monitored to make sure the G2 peak has the double intensity of the G1 peak. Moreover, G2 and S phase identity have been ascertained comparing with X-ray treated samples in which their strong reduction is expected. Two independent experiments were performed for each experimental dataset to verify the consistency of the results. The non-parametric, two-tailed Mann–Whitney *U*-test was applied across each experiment (*n* = 5, each obtained by pooling ten different animals) to analyse the statistical significance of differences between each treatment group and the untreated control group. Differences were considered statistically significant with a *p*-value below 0.05.

#### 4.5.4. Quantification of H3p stained cells

In cytometry experiment H3p-positive cells were identified by comparing control untreated samples with X-ray treated samples in which, absence of mitosis, has been widely documented. This allowed setting a gate for H3p-positive cells that has been applied to all the other datasets. Two independent experiments were performed for each experimental dataset to verify the consistency of the results. The non-parametric, two-tailed Mann–Whitney *U*-test was applied across each experiment (*n* = 5, each obtained by pooling ten different animals) to analyse the statistical significance of differences between each treatment group and the untreated control group. Differences were considered statistically significant with a *p*-value below 0.05. In whole mount fluorescent immunostaining the amount of H3p-positive cells was quantified, using the find maxima function of the ImageJ software, in the full thickness of the anterior part of the animal to quantify general changes in mitotic activity, or in merged images including only the most dorsal focal planes, from the epidermis to the opening of the anterior gut branch lumen, in way to quantify mitotic activity in a region enriched in DMC cells. To this aim, in these merged images we quantified the H3p-positive cells in a box along the midline arbitrary drawn in blind by two independent operators. The number of mitosis was normalized in accordance with animal size dividing by the surface area drawn at the boundary of the scanned animal. Two independent experiments were performed for each experimental dataset to verify the consistency of the results. The non-parametric, two-tailed Mann–Whitney *U*-test was applied across each experiment (*n* = 10) to analyse the statistical significance of differences between each treatment group and the untreated control group. Differences were considered statistically significant with a *p*-value below 0.05. Number of mitoses in the DMC was also quantified by manual counting H3p-positive cells in the DMC territory demarcated by DjPiwiA labelling. The non-parametric, two-tailed Mann–Whitney *U*-test was applied across each experiment (*n* = 5) to analyse the statistical significance of differences between each treatment group and the untreated control group. Differences were considered statistically significant with a *p*-value below 0.05.

#### 4.5.5. Quantification of DjPiwiA-positive cells

In way to set appropriate gates for the quantification of DjPiwiA-positive cells we compared plots of samples stained with the secondary antibody alone, with those of samples stained with DjPiwiA antibody. This, taking in mind that according to literature S phase cells are all positive for DjPiwiA, allowed us to set gates for discriminating positive from negative cells. The non-parametric, two-tailed Mann–Whitney *U*-test was applied across each experiment (*n* = 5, each obtained by pooling ten different animals) to analyse the statistical significance of differences between each treatment group and the untreated control group. Differences were considered statistically significant with a *p*-value below 0.05.

## Data Availability

All data and protocols are available in the manuscript in the material and methods section. The data are provided in electronic supplementary material [63].
